# Normalization of Exhaled Carbonyl Compounds After Lung Cancer Resection

**DOI:** 10.1016/j.athoracsur.2016.04.068

**Published:** 2016-10

**Authors:** Erin M. Schumer, Matthew C. Black, Michael Bousamra, Jaimin R. Trivedi, Mingxiao Li, Xiao-An Fu, Victor van Berkel

**Affiliations:** a Department of Cardiovascular and Thoracic Surgery, University of Louisville, Louisville, Kentucky; b Department of Chemical Engineering, University of Louisville, Louisville, Kentucky

## Abstract

**Background:**

Quantitative analysis of specific exhaled carbonyl compounds (ECCs) has shown promise for the detection of lung cancer. The purpose of this study is to demonstrate the normalization of ECCs in patients after lung cancer resection.

**Methods:**

Patients from a single center gave consent and were enrolled in the study from 2011 onward. Breath analysis was performed on lung cancer patients before and after surgical resection of their tumors. One liter of breath from a single exhalation was collected and evacuated over a silicon microchip. Carbonyls were captured by oximation reaction and analyzed by mass spectrometry. Concentrations of four cancer-specific ECCs were measured and compared by using the Wilcoxon test. A given cancer marker was considered elevated at 1.5 or more standard deviations greater than the mean of the control population.

**Results:**

There were 34 cancer patients with paired samples and 187 control subjects. The median values after resection were significantly lower for all four ECCs and were equivalent to the control patient values for three of the four ECCs.

**Conclusions:**

The analysis of ECCs demonstrates reduction to the level of control patients after surgical resection for lung cancer. This technology has the potential to be a useful tool to detect disease after lung cancer resection. Continued follow-up will determine whether subsequent elevation of ECCs is indicative of recurrent disease.

Drs Bousamra, Fu, and van Berkel disclose a financial relationship with Breath Diagnostics, Inc.

In 2015, it was estimated that 221,200 Americans would be diagnosed with lung cancer. Lung cancer remains the leading cause of cancer death in the United States and represents 13% of all cancer diagnoses [1]. Despite improvements in surgical procedures, chemotherapy, and radiation therapy, overall survival has remained poor, with a 5-year survival of 18.0% [2]. Survival in other common cancers, specifically breast, prostate, and colon, have seen more drastic improvements, which can be partly attributed to earlier detection, as effective screening technology and modalities are developed 3, 4. However, these screening techniques are not uniformly effective and can result in overdiagnosis of benign findings with risk of unnecessary morbidity 3, 5.

The National Lung Screening Trial, published in 2011, found a 6.7% overall survival benefit for patients at high risk of lung cancer, who underwent yearly screening computed tomography (CT) scans [6]. Lung cancer mortality was also reduced by 20%. The study was able to detect a high proportion of early cancers (49% stage IA), allowing for intervention with curable intent. The National Lung Screening Trial provided a positive test in 24.2% of patients; however, 94% of these were falsely positive [7]. These false positives have led to further investigation with multiple modalities, including radiographic and sometimes invasive testing, resulting in risk of unnecessary major complications for potential benign disease [8]. This method of screening will substantially contribute to health care cost, with estimates in the range of billions of dollars annually [9]. It may be further limited by patient compliance, which is uncertain. These shortcomings provide an opportunity for improvements in lung cancer detection and the development of alternative strategies.

Similarly, a diagnostic method that could also contribute to surveillance after resection would be ideal. Even in early-stage lung cancer, recurrence occurs in 20% of patients, and up to 7% experience a second primary recurrence [10]. Currently, no universally agreed on surveillance guidelines exist for interval frequency or method type. Hanna and colleagues [11] found that CT scan is overwhelmingly superior to chest x-ray (CXR) in the sensitivity of detecting recurrence (94% versus 21%), but these results have not been reproduced in other studies. Crabtree and colleagues [12] showed a shorter time interval to detection of a successive malignancy when using surveillance CT scan over CXR, but they also showed no difference in detection of malignancy overall, 5-year survival, or rate of treatment for curative intent. This has led to various guidelines, as published by the National Comprehensive Cancer Network, the American College of Chest Physicians, and the American Association of Thoracic Surgeons, among others. These guidelines suggest some combination of history and physical examination and imaging method, usually CT with the option for substituting CXR, at different intervals for different postoperative years [13].

The analysis of exhaled breath is a promising noninvasive diagnostic tool for distinguishing benign from malignant pulmonary disease [14]. This technology has the potential to also provide a mechanism for surveillance after resection of patients diagnosed with lung cancer. This study examined the trends of exhaled carbonyl compounds (ECCs) after resection of malignant nodules, to see if the detected levels of ECCs would normalize to background levels after resection.

## Material and Methods

### Collection of Breath Samples

The University of Louisville Institutional Review Board approved the research protocol for collection of exhaled breath samples. All study subjects signed informed consent before providing breath samples. A single exhalation of 1 L of breath was collected with a Tedlar bag (Sigma-Aldrich, St Louis, MO) from each subject. Breath samples were collected from 222 subjects. Lung cancer was pathologically confirmed in 31 patients. Benign disease was confirmed pathologically in 4 patients. A breath sample after resection was collected in a similar manner. Some patients provided more than one postresection sample, but all 31 patients provided at least one sample. The time interval between collections was varied. The remaining 187 patients served as the control group that consisted of healthy patients without known lung disease, which included nonsmokers, active smokers, and former smokers.

### Silicon Chip and Mass Spectrometry

As previously described, the silicon microchips consisted of an array of micropillars fabricated from silicon wafers [15]. A quaternary ammonium compound, 2-(amino-oxy)-N, N, N trimethylethanammounium (ATM) iodide, was used to coat the surfaces of the micropillars. Through electrostatic and hydrogen bonds, the compound adsorbs to the silicon dioxide surfaces of the micropillars. By means of oximation reactions, the ATM selectivity traps carbonyl compounds in exhaled breath with capture efficiencies of 98% or greater.

The process of carbonyl compound capture from exhaled breath has been previously described [16]. In summary, exhaled breath collected in 1-L Tedlar bags was drawn through the silicon microreactor chip with the use of an applied vacuum. Next, ATM iodide adducts on the microreactor chip were eluted with methanol from a slightly pressurized small vial; recovery of ATM adducts is 99%. The eluted solution was directly analyzed by Fournier transform-ion cyclotron resonance-mass spectrometry. A known amount of deuterated acetone that had completely reacted with ATM (ATM-acetone-d6) was added to the eluted solution as an internal reference. The concentrations of all carbonyl compounds in the exhaled breath were determined by comparison of relative abundance with that of added ATM-acetone-d6.

The entire study population consists of analysis that used two different microchips with different densities of micropillars. Subanalysis of these groups did not reveal any meaningful difference between the two microchips.

### Data Analysis

Four distinct carbonyl cancer markers (2-butanone, 3-hydroxy-2-butanone, 2-hydroxyacetaldehyde, and 4-hydroxyhexanal) were used for this analysis; previous studies have reported elevated levels of these markers in cancer patients [14]. Control values were established in the aforementioned 187 healthy patients. Some patients that were included in the previously reported cancer data set are included in this study after contributing postresection samples.

From previous studies, a positive carbonyl marker was defined at 1.5 or more standard deviations greater than the mean of the control population [14]. A positive breath test was defined as one or more positive carbonyl marker, with a given patient having between zero and four elevated carbonyl cancer markers. The concentrations of these carbonyl markers were compared and represented graphically for breath samples both before and after resection in patients with pathologically confirmed cancer. Similarly, postresection samples were compared with the established concentrations of the control population. Statistical significance was determined with a nonparametric statistical hypothesis test. Descriptive and univariate analyses were performed with SPSS, version 22 (IBM, Armonk, NY).

## Results

A total of 35 patients had samples before and after resection; 31 had lung cancer and 4 had benign pulmonary disease. The 31 lung cancer patients underwent surgical resection and had at least one breath sample before and after resection. Characteristics of the lung cancer cohort are shown in Table 1.

**Fig 1:**
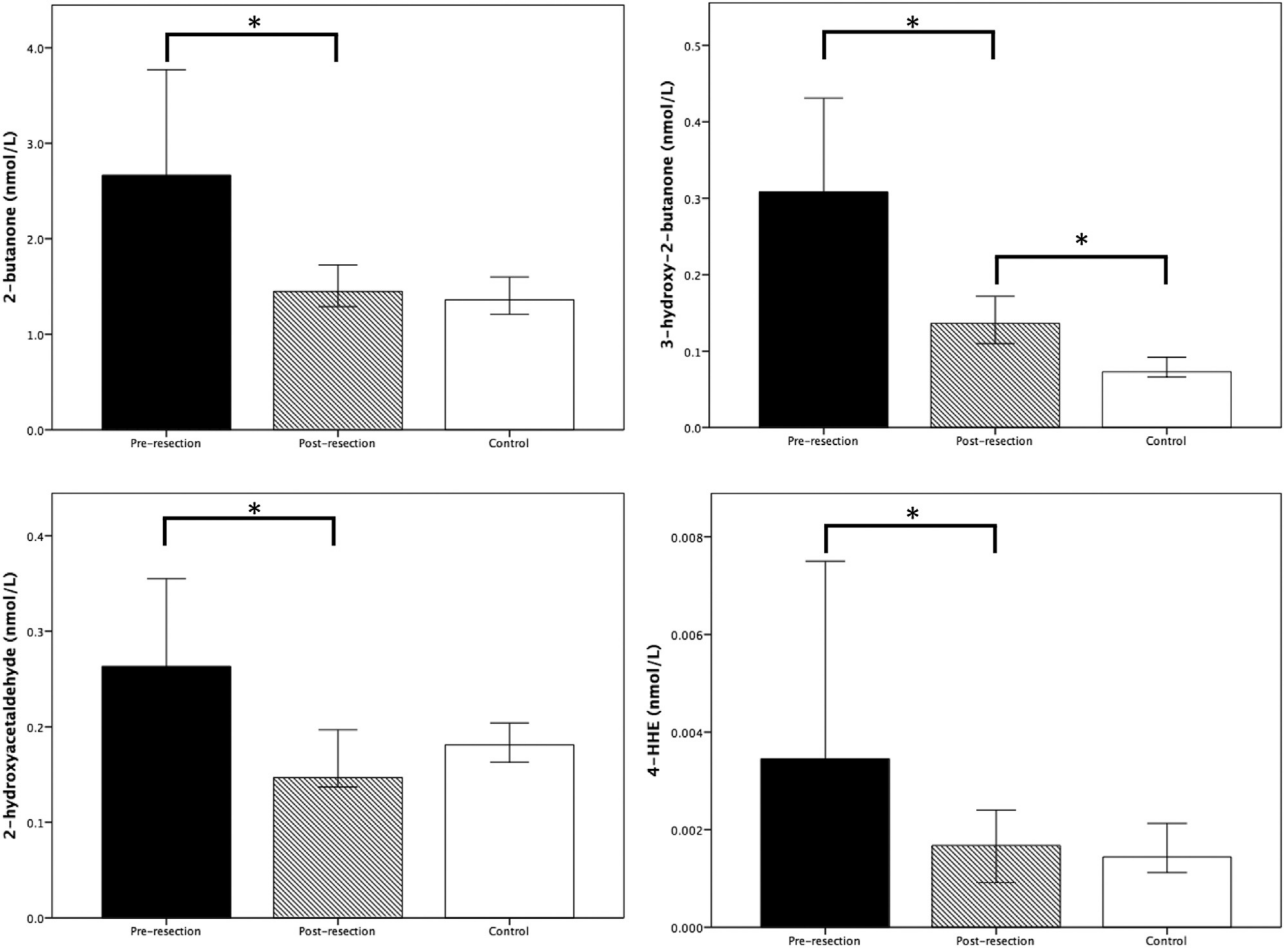
Median concentrations for carbonyl compounds before and after resection in patients who underwent lung transplantation compared with median control values. Error bars represent 95% confidence interval. Concentrations are in nmol/L. *p < 0.05. (4-HHE = 4-hydroxyhexanal.)

**Fig 2:**
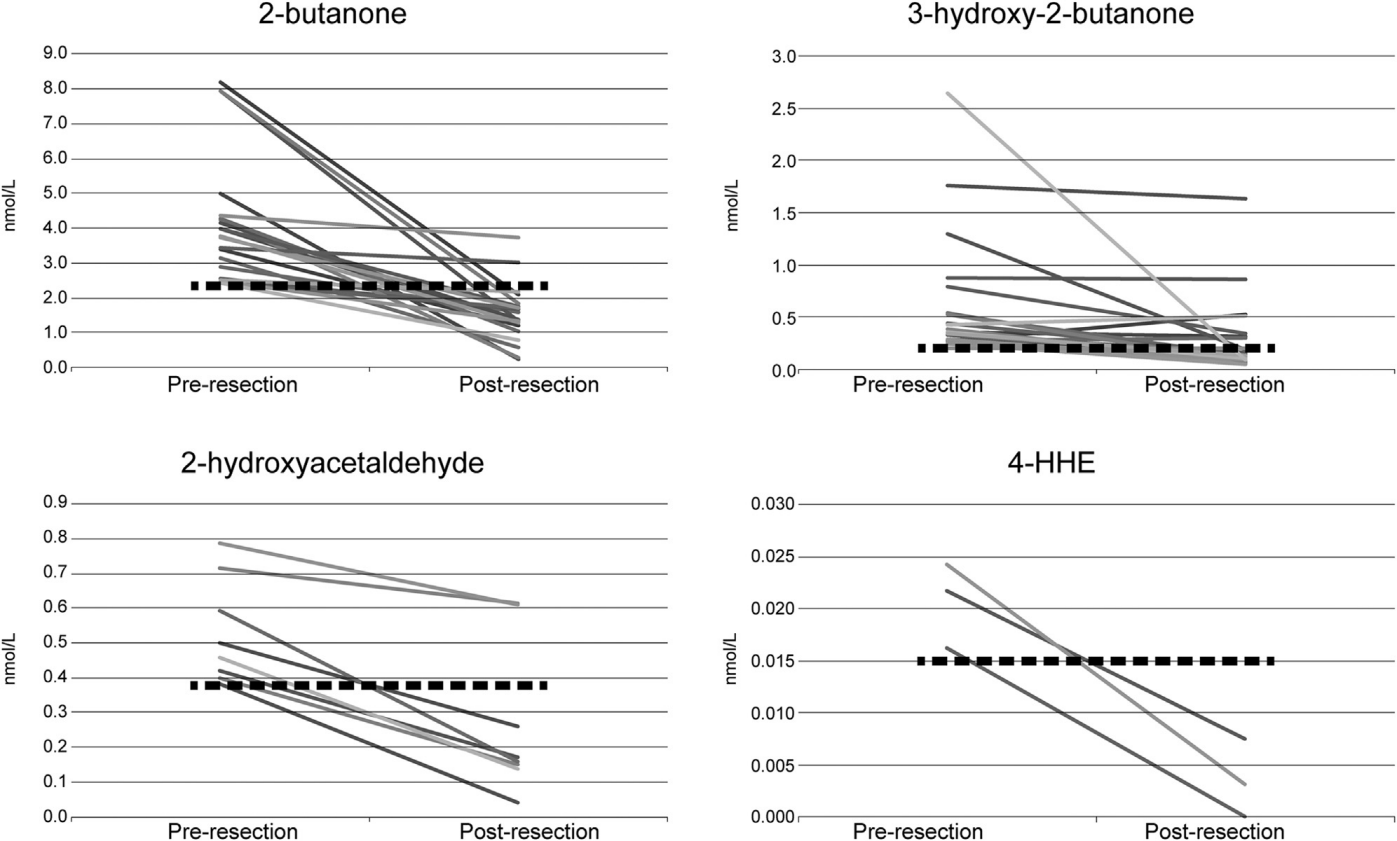
Values for 2-butanone, 3-hydroxy-2-butanone, 2-hydroxyacetaldehyde, and 4-hydroxyhexanal (4-HHE) before and after resection when the value before resection is elevated. The dashed line is the threshold for an elevated carbonyl compound. Concentrations are in nmol/L.

The lung cancer patients were analyzed separately. Median values before and after resection were compared for the four ECCs. Median concentrations were significantly lower for all cancer markers after resection and are shown in Figure 1. Postresection values were then compared with the control patients (n = 187) and are shown for comparison in Figure 1. No significant differences were found in the median concentrations of 2-butanone, 2-hydroxyacetaldehyde, or 4-hydroxyhexanol between postresection and control concentrations. The postresection concentration of 3-hydroxy-2-butanone was, however, significantly higher than that of the control group. Figure 2 demonstrates the concentrations before and after resection for lung cancer patients for individual carbonyls that were elevated before resection.

Four patients with benign pulmonary disease underwent pulmonary resection. Histologic examination was positive for granulomatous disease in all patients. Three patients had zero ECCs both before and after resection. One patient had three elevated ECCs before resection that normalized after resection.

## Comment

Our group has previously demonstrated the ability of analysis of ECCs to detect lung cancer [15], to distinguish from benign pulmonary disease [16], and to potentially serve as a screening examination with the use of four compounds, 2-butanone, 3-hydroxy-2-butanone, 2-hydroxyacetaldehyde, and 4-hydroxyhexanal [14]. This current study of 31 lung cancer patients treated surgically demonstrates a significant decrease in the concentration of four ECCs after pulmonary resection. In addition, three of four ECCs normalized to the level of the control population after cancer resection, further confirming the relation of these carbonyl markers to cancer.

Although screening high-risk patients for lung cancer with low-dose CT scanning has been shown to reduce both all-cause and lung cancer-specific mortality [6], post-treatment surveillance guidelines for lung cancer are variable and based on a low level of evidence. In addition, it is unclear whether the use of CT scan for surveillance even affects mortality from lung cancer [12]. The National Comprehensive Cancer Network surveillance guidelines recommend a chest CT scan every 6 to 12 months for 2 years, followed by annual chest CT scans. Not only are these recommendations based on a low level of evidence (2A), but the high frequency of CT scans is expensive 17, 18, results in false positives that likely lead to unnecessary testing and potential complications [19], and exposes lung cancer patients to a high amount of radiation. In addition, it has been found that patient compliance with surveillance is highly variable, depending on socioeconomic status, and may be related to the inconvenience of frequent CT scans 20, 21. Surveillance with breath analysis has the potential to offer these patients a less-expensive, more-convenient test that avoids radiation exposure.

Although ECCs are clearly associated with malignancy, the mechanism of their formation is still unknown. The rapid normalization of three of the four compounds after resection provides strong evidence that they are directly produced by the tumor environment. Several hypotheses exist about ECC production, including altered metabolism by cytochrome p450 enzymes and production of reactive oxygen species in the tumor microenvironment that produce the carbonyl compounds by reactions with fatty acids 22, 23. The lack of consistent normalization of 3-hydroxy-2-butanone after resection suggests that this compound may be tied more closely to inflammation associated with tumors rather than the tumors themselves and, thus, may remain elevated longer after resection. Further studies to identify the mechanisms involved in the production of carbonyls, and their relation to other cancers, will answer these questions.

The study has limitations. The number of patients in the study population is small. A larger number of lung cancer patients with multiple breath samples must be recruited to validate the findings presented in the current study. Furthermore, samples were collected at various time lengths. A standardized collection method would yield an increased number of samples and the ability to possibly delineate changes in carbonyl composition over time. Finally, this study population did not include any patients with recurrent lung cancer. To truly determine the role of breath analysis in surveillance of lung cancer, recurrent lung cancer must be detected and compared with the ability of CT scanning to detect recurrent lung cancer.

In conclusion, breath analysis of ECCs demonstrates normalization in lung cancer patients after pulmonary resection. Thus, breath analysis may be useful as a potential tool after lung cancer treatment. Further studies will reveal the ability of breath analysis to detect recurrent lung cancer and may determine the role of breath analysis in lung cancer surveillance after resection.

## Figures and Tables

**Table 1:** Demographic Characteristics for the Lung Cancer Patients

Characteristic	Value
Sex, male	14 (45.2)
Age, years	68 (39–84)
Time between samples, days	287 (21–1106)
Smoking	
Current	12 (38.7)
Former	16 (51.6)
None	3 (9.7)
Stage	
0	1 (3.2)
IA	14 (45.2)
IB	9 (29.0)
IIA	2 (6.5)
IIB	4 (12.9)
IV	1 (3.2)
Histology	
Adenocarcinoma	15 (48.4)
Squamous cell carcinoma	12 (38.7)
Other	4 (12.8)
Total	31

Values n (%) or median (range).

## Discussion

**DR DANIEL L. MILLER** (Marietta, GA): That was an excellent presentation. Was there any change in effect related to if the patient was still smoking or not from the preoperative to the postoperative time period?

**DR SCHUMER:** We did not look at that specifically, but, in previous studies, we have compared nonsmokers with smokers, and we did not find a difference between those two groups in three of the four compounds. And it was the same compound, 3-hydroxy-2-butanone, that did show a slight difference.

**DR MILLER:** And then in regard to normalization back to below, what was that time period, 3 months, 6 months? When did that occur?

**DR SCHUMER:** It was highly variable, and, again, we have a low number of patients in the group, so it was very dependent. Some would normalize shortly after resection, but in the patient example that I showed, one of the compounds did not normalize until 2 years after resection that had been elevated before resection.

I think we would need to look at more patients to determine what time frame that these compounds do normalize and whether resection actually does affect increase in the compound because there may be some sort of inflammatory component too.

**DR MILLER:** In the squamous cell patients, maybe it was from the squamous dysplasia or something going on in the airway that did not allow them to normalize. Thank you.

**DR MATTHEW G. BLUM** (Colorado Springs, CO): I had two questions, and one of them was, do you have any mechanism for why cancers or cancer-related tissue would be producing these particular compounds?

And the other is, did you look at levels of these compounds in patients who underwent resection for benign disease to see if that also shifted their levels down even though they started with relatively normal levels?

**DR SCHUMER:** To answer your second question first, we have four patients that have benign disease that did undergo resection. Three of the four patients had zero elevated markers before resection. After resection, they remain not elevated.

There is one patient that did have three elevated markers before resection, and this did go down to zero. But, again, we would have to look at a lot more patients.

**DR BLUM:** How about the other question?

**DR SCHUMER:** Oh, to answer your second question.

**DR BLUM:** Did you have any mechanism there?

**DR SCHUMER:** Really, we do not have a good idea of the mechanism behind the elevation of these compounds. There have been some papers by other groups that suggest P450 may be involved in metabolism, but we do not have a good explanation at this point.

**DR JOSEPH B. SHRAGER** (Stanford, CA): One question would be, is there a difference between adenocarcinoma and squamous with these markers?

And then the other one is, how about aggressiveness of the tumors? For example, do they correlate with SUVmax or size of the tumor or that sort of thing?

You did not really show us here, sensitivity, specificity, positive predictive value. Are those sorts of statistics available in a previous study?

**DR SCHUMER:** Previously, we have looked at a range of sensitivity and specificity, based on the cutoff level that we use and the number of markers that are elevated. If you use one marker at 1.5 standard deviations, which is what we used for this study, it is about a 94% sensitivity. And, of course, that will go down as the number of markers that you choose goes up.

As far as the difference between adenocarcinoma and squamous cell carcinoma, no, we have not looked at that specifically, but we have seen across the board all histologies, including small cell lung cancer, that carbonyls are elevated. But we have not compared the specific histologies.

**DR SHRAGER:** The more aggressive tumors by SUVmax or that sort of thing, do they make more of this stuff?

**DR SCHUMER:** So the answer is yes. In one of our previous papers, we did look at PET scans compared with breath analysis, and so for patients with later-stage disease, they are more likely to have elevated markers than patients with early-stage disease.

**DR DANIEL L. MILLER** (Marietta, GA): Can I ask one more question? What is the cost of the test?

**DR SCHUMER:** The cost is cheap. There are up-front costs involved, so you have to have a mass spectrometer, for example.

**DR MILLER:** How much are those running on Amazon these days?

**DR SCHUMER:** I do not have a good answer to that. But the test itself is cheap. It is around $30.

**DR MILLER:** Thank you. Nice job.
